# Sperm Cryodamage in Ruminants: Understanding the Molecular Changes Induced by the Cryopreservation Process to Optimize Sperm Quality

**DOI:** 10.3390/ijms21082781

**Published:** 2020-04-16

**Authors:** Patricia Peris-Frau, Ana Josefa Soler, María Iniesta-Cuerda, Alicia Martín-Maestro, Irene Sánchez-Ajofrín, Daniela Alejandra Medina-Chávez, María Rocío Fernández-Santos, Olga García-Álvarez, Alejandro Maroto-Morales, Vidal Montoro, J. Julián Garde

**Affiliations:** SaBio IREC (CSIC-UCLM-JCCM), ETSIAM, Campus Universitario s/n, 02071 Albacete, Spain; Patricia.Peris@uclm.es (P.P.-F.); m.iniestacuerda@gmail.com (M.I.-C.); Alicia.MartinMaestro@uclm.es (A.M.-M.); Irene.SSanchez@uclm.es (I.S.-A.); Daniela.Medina@uclm.es (D.A.M.-C.); mrocio.fernandez@uclm.es (M.R.F.-S.); garciaalvarez.olga@gmail.com (O.G.-Á.); jandromaroto@hotmail.com (A.M.-M.); Vidal.montoro@uclm.es (V.M.); Julian.garde@uclm.es (J.J.G.)

**Keywords:** ruminant species, sperm cryopreservation, sperm cryodamage, proteomics

## Abstract

Sperm cryopreservation represents a powerful tool for livestock breeding. Several efforts have been made to improve the efficiency of sperm cryopreservation in different ruminant species. However, a significant amount of sperm still suffers considerable cryodamage, which may affect sperm quality and fertility. Recently, the use of different “omics” technologies in sperm cryobiology, especially proteomics studies, has led to a better understanding of the molecular modifications induced by sperm cryopreservation, facilitating the identification of different freezability biomarkers and certain proteins that can be added before cryopreservation to enhance sperm cryosurvival. This review provides an updated overview of the molecular mechanisms involved in sperm cryodamage, which are in part responsible for the structural, functional and fertility changes observed in frozen–thawed ruminant sperm. Moreover, the molecular basis of those factors that can affect the sperm freezing resilience of different ruminant species is also discussed as well as the molecular aspects of those novel strategies that have been developed to reduce sperm cryodamage, including new cryoprotectants, antioxidants, proteins, nanoparticles and vitrification.

## 1. Introduction

Sperm cryopreservation has become an essential tool for the long-term preservation of genetically superior males, relevant transgenic lines and endangered species [1,2]. Moreover, cryopreservation facilitates the distribution of semen over distance, which has greatly contributed to the expansion of reproductive technologies such as artificial insemination and in vitro fertilization worldwide [3].

However, spermatozoa are subjected to drastic changes in temperature, ice crystal formation and diverse types of stresses (physical, chemical, osmotic and oxidative) during the cryopreservation process, which severely compromise sperm quality and fertility [4,5,6]. In addition, differences between species in terms of sperm size, shape and lipid–protein composition denote that the cryopreservation process is not equally efficient among all species [7]. Bulls, rams, horses and boars have been reported to generate more cryosensitive sperm than humans, rabbits, cats and dogs [8]. Aside from interspecies variability, the success of cryopreservation is also affected by many other factors like cooling–thawing rates, type of extender or cryoprotectants, sperm source (epididymal or ejaculate sperm), seasonal variations and even by inter- or intra-individual variations [9,10]. Thereby, increasing our knowledge about the molecular modifications suffered by sperm through the cryopreservation process will aid the optimization of the current freezing–thawing protocols for a better preservation of sperm functionality and fertility. In recent years, the use of different omics technologies in the field of sperm cryobiology has led, among other things, to a greater understanding of those structural and functional changes experienced by sperm during the freezing–thawing process as well as the identification of relevant freezability biomarkers [4,11,12].

Novel findings about the effects of sperm cryopreservation have led to the development of new techniques and methods of cryopreservation, where different proteins, antioxidants and cryoprotective agents are being incorporated into the freezing medium for increasing sperm cryosurvival. Such improvements, however, have not yet reached the desired level because many sperm still lose their viability after cryopreservation [13]. Therefore, the enhancement of sperm cryopreservation outcomes remains a major challenge, especially in ruminant animals, which include cattle, sheep, goat, buffalo, deer and other wild species. This group of mammals, which is widespread around the world, constitutes important food sources (meat and milk) and contributes to maintaining a sustainable agriculture due to these mammals’ ability to feed on those fibrous vegetables or by-products that cannot be used as human food [14].

This review explores how those molecular elements (proteins, lipids, ARNs, epigenetic marks, etc.) altered by sperm cryopreservation can affect the different structures or functions of ruminant sperm. In addition, the molecular mechanisms involved in the sperm freezing resilience of different ruminant species are further discussed as well as the molecular aspects of those new approaches to improve the sperm cryopreservation outcomes in ruminants, such as new cryoprotectants, antioxidants, proteins, nanoparticles and vitrification.

## 2. Unraveling How the Molecular Damage Caused by the Freezing–Thawing Process Affects Sperm Structure and Function

**Figure 1:**
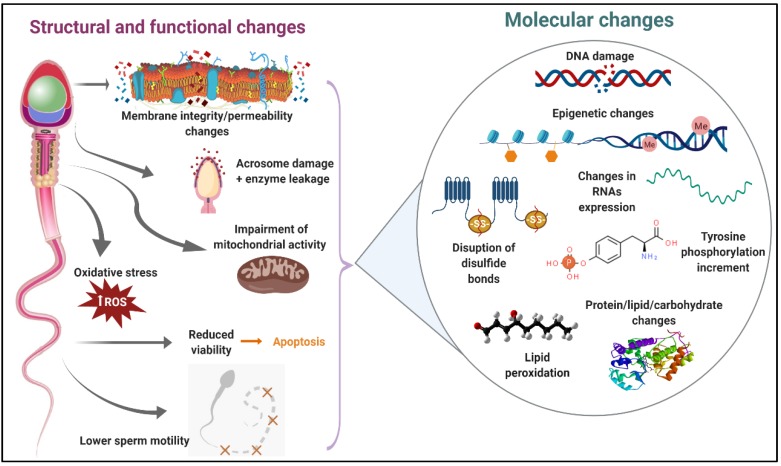
Main consequences of sperm cryodamage in ruminants. During the cryopreservation process, ruminant sperm suffer several structural and functional damages, which are probably the result of different molecular changes. This figure summarizes those structural, functional and molecular changes produced during the freezing–thawing procedure.

In general, sperm cryopreservation has been reported to induce an increase in plasma membrane fluidity–permeability, overproduction of reactive oxygen species (ROS), reduction of acrosome integrity, impairment of mitochondrial membrane potential and lower sperm motility in bull [15,16,17,18,19], buffalo [20,21,22], buck [23,24,25], ram [26,27,28,29,30] and red deer [31]. Molecular studies during sperm cryopreservation offer the possibility of recognizing those specific elements (proteins, lipids, ions, carbohydrates, etc.) altered by the freezing–thawing process that are in part responsible for the structural and functional changes observed in cryopreserved sperm (Figure 1).

In this sense, understanding the molecular modifications inflicted by the freezing–thawing process is essential to diminish or prevent cryodamage. Owing to the reduced [32], if not seemingly absent, [33,34] transcriptional and translational activity in mature sperm, proteomics studies represent the best option for investigating the molecular mechanisms regulating sperm functionality [35]. Moreover, it is also important to study the impact of cryopreservation on sperm RNAs transcripts since some of them are delivered to the oocyte participating in fertilization and embryo development, while others are involved in capacitation, motility, metabolism and other relevant sperm functions [36].

### 2.1. Molecular Changes in the Sperm Plasma Membrane during Cryopreservation

One of the first structures affected by cryopreservation is the sperm plasma membrane [2]. In ruminants, the sperm plasma membrane contains high levels of unsaturated phospholipids and low levels of cholesterol. Such composition, particularly the lower content of cholesterol, declines the resistance of sperm to the freezing–thawing process [37]. During freezing, phospholipids undergo a redistribution across the membrane, and some of them change from liquid to gel state earlier than others due to structural differences, resulting in a lipid phase separation [8]. In consequence, the lipid–protein interactions required for a proper membrane activity are disturbed [9], and some sperm surface proteins as well as membrane proteins are lost or translocated with the consequent loss of their function. For example, proteins involved in capacitation, sperm–oocyte interaction and gamete fusion, such as TCP1, LOC101123268, RPN1, P25b, HEXB, CSNK1G2, ICA, LOC101123216, ADAM2 and TIMP-2, decreased in abundance in ram, gazelle and bull sperm after cryopreservation [38,39,40,41], while another protein associated with fertilization, HSP70, was lost in buffalo sperm [42]. Other proteins involved in transport, membrane stabilization and protection against lipid peroxidation or cold-shock, such as GLUT, CLU, BSP5, BSP1, aSFP, HSPA4L, TRAP1, GPX4 and GPX5 also decreased in abundance in these species along with antiapoptotic and decapacitating proteins (CSNK2A2 and Spermadhesin Z13) [38,40,43,44].

Cryopreservation also induces significant changes in the distribution or abundance of those proteins that act as ROS scavengers. Relevant antioxidant enzymes such as glutathione peroxidase (GPx), glutathione reductase (GR) and superoxide dismutase (SOD) were redistributed on ram sperm surface following cryopreservation [45]. These findings, together with the reduced antioxidant activity of SOD and reduced glutathione (GSH) observed in bull and ram sperm after cryopreservation, could explain in part the increased susceptibility of frozen–thawed sperm to suffer lipid peroxidation and oxidative damage [45,46]. Conversely, other studies showed that antioxidant enzymes such as SOD2 and PRDX5 increased after cryopreservation in bull sperm [44,47,48]. Whether this increment is the result of a protein reorganization like in ram sperm, or, on the contrary, if it really reflects a higher activity of these enzymes to protect sperm against oxidative attack, needs to be further verified.

The antioxidant defense of sperm is quite scarce and basically depends on the antioxidant capacity of seminal plasma [49]. Seminal plasma contains enzymatic (GPx, SOD and CAT) and non-enzymatic antioxidants (GSH, pyruvate, urate, ascorbic acid, α-tocopherol, taurine and hypotaurine); however, its protective effect against oxidative stress significantly decreases when semen is diluted in the freezing medium prior to cryopreservation [46].

**Figure 2:**
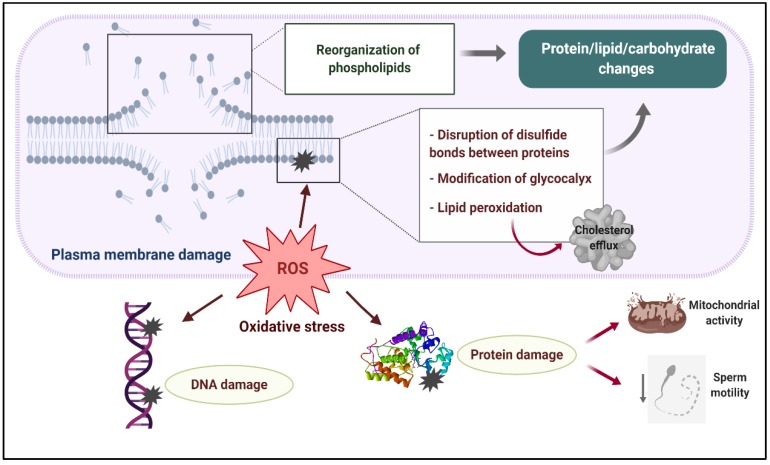
Plasma membrane damage during sperm cryopreservation and its relationship with oxidative stress. A reorganization of sperm membrane phospholipids takes places during freezing–thawing, altering lipid–protein, lipid–carbohydrate and protein–carbohydrate interactions which are necessary for proper membrane activity. Excessive production of reactive oxygen species (ROS) leads to major protein, lipid and carbohydrate changes in the sperm membrane due to the reduction of disulfide bonds between membrane proteins, peroxidation of membrane phospholipids and modifications of the sperm glycocalyx. As a result, the sperm membrane becomes fragile and its semipermeable property is lost. Overproduction of ROS during sperm cryopreservation may also cause DNA damage and impair several axonemal and mitochondrial proteins, which negatively affect mitochondrial activity and axonemal integrity, resulting in the loss of sperm motility.

Disturbances in the sperm antioxidant system during cryopreservation and the activation of L-Amino acid oxidase in dead or defective cryopreserved sperm significantly contribute to the increased ROS production detected in ruminant sperm after freezing–thawing, the sperm plasma membrane being the primary site where ROS-induced damage is manifested (Figure 2) [3,17,28,50]. Excessive generation of ROS during cryopreservation leads to major protein, lipid and carbohydrate changes in the sperm membrane due to the reduction of disulfide bonds between membrane proteins [51], peroxidation of membrane phospholipids and modifications of the sperm glycocalyx [12]. Ruminant sperm are particularly susceptible to suffer lipid peroxidation due to the higher content of polyunsaturated phospholipids in the plasma membrane [2]. Overproduction of ROS triggers the oxidative attack of polyunsaturated phospholipids, resulting in the formation of malondialdehyde (MDA), 4-hydroxynonenal (4HNE), acrolein and other toxic byproducts which may attack other polyunsaturated phospholipids [49,50]. As a consequence of this peroxidative damage, some cholesterol, phosphatidylethanolamine and phosphatidylcholine molecules are released [12]. This lipid peroxidative attack, together with the conformational changes that the plasma membrane experience during cryopreservation, have a negative impact on membrane integrity, destroying its semipermeable properties [1,15,18].

### 2.2. Molecular Disturbances in Sperm Energy Metabolism and Motility during Cryopreservation

During sperm cryopreservation, the consequences of oxidative damage are numerous. Besides sperm membrane damage, oxidative stress disrupts mitochondrial activity, promotes the efflux of intracellular enzymes and impairs several axonemal proteins with the consequent loss of sperm motility [52]. Lipid peroxidation derivatives, such as 4HNE and MDA, can diffuse into other cellular compartments and bind to protein nucleophiles, forming adducted proteins [12,50]. Adduct formation decreases and even disrupts protein function after altering protein structure. Aitken et al. [53] showed that axonemal proteins, such as dyneins, tubulins and heat-shock proteins, as well as mitochondrial proteins, like succinate dehydrogenase and ATP synthases, are common targets of these lipid peroxides. Protein adduction in the first case disturbs cytoskeleton structure, while in the second case it adversely affects the activity of electron transport chain. The electron transport chain is an essential component of mitochondrial oxidative phosphorylation involved in ATP production. Consequently, disturbances in the electron transport chain due to protein adduction lead to ATP depletion and a self-perpetuating cycle of ROS production that eventually initiates the apoptotic cascade [50]. Mostek et al. [54] reported that several proteins involved in cytoskeletal organization (Ropporin-1 (ROPN1), outer dense fiber protein 2 (ODF2), capping protein beta3 isoform, actin-related protein T2 (ACTRT2), actin-related protein M1) and energy metabolism (NADH dehydrogenase, isocitrate dehydrogenase, triosephosphate isomerase) underwent oxidative modifications via carbonylation during cryopreservation in bull sperm. However, more studies are needed to better understand the role of protein carbonylation in sperm function.

In ruminants, two main metabolic pathways, oxidative phosphorylation and glycolysis, produce the energy required to maintain sperm motility in the form of ATP [55]. Comparative proteomics studies between fresh and cryopreserved sperm revealed that freezing–thawing procedures alter the abundance of several enzymes implicated in oxidative phosphorylation and glycolysis in ram, bull and gazelle sperm [38,43,44,47]. Among them, different ATP synthases, COX5B, AK1, NDUFV2, ODPB2, ACO2 and NDPK7 were some of those proteins related to oxidative phosphorylation, while different hexokinases, GPI, ALDOA, GAPDH5, PGK2, PGAM2, PKM2 and TPI were some of those proteins related to glycolysis. Although the cause of these proteomics variations is yet to be resolved, some authors proposed diverse mechanisms to explain such differences, which include protein degradation, oxidation, tyrosine phosphorylation and translocation [44,54]. Modifications of mitochondrial and glycolytic proteins during cryopreservation could be in part responsible for the reduced motility of frozen–thawed sperm due to the lack of ATP production. Diverse studies demonstrated that the loss of sperm motility during freezing–thawing was associated with the impairment of mitochondrial activity [19,21,22], and a similar relationship between glycolysis and sperm motility was also suggested [38,43].

Besides the negative effect of sperm cryopreservation on metabolic enzymes, cytoskeletal proteins are temperature-sensitive. This fact explains why some cytoskeletal proteins decrease in abundance (TEKT4, ODF2, ROPN1, ACTRT2, ACTL7B and actin) or change their distribution (F-actin, actin and β-dystrobrevin) during freezing–thawing in bull, ram, gazelle and buffalo sperm [38,43,47,48,56,57]. Such findings have significant repercussions on sperm motility since cytoskeletal proteins are involved in the maintenance of axoneme integrity. In addition, another protein (HSP90) localized in the flagellum and related to sperm structure and ATP metabolism also decrease in abundance during cryopreservation [58]. Consequently, the reduced motility of cryopreserved sperm is probably the result of both axonemal protein damage and alterations in energy availability due to enzyme modifications.

### 2.3. Molecular Changes in the Sperm Chromatin during Cryopreservation

Sperm with damaged DNA can complete the fertilization process; however, embryo development can be seriously interrupted or altered once the embryo genome is activated at the 4- or 8-cell stage due to the transcription of damaged paternal genes [59,60]. Contradictory results have been found regarding the effect of cryopreservation on sperm DNA integrity. While some authors reported that cryopreservation impaired sperm DNA integrity [15,16,61], others reported that it was unaffected immediately after thawing but that significant DNA damage appeared during the subsequent incubation [29,62,63,64]. Although the mechanisms involved in sperm DNA damage are yet unclear, oxidative and mechanical stress appear to be the main causes during cryopreservation [16,65].

A number of tests are currently available to evaluate sperm DNA fragmentation in ruminants. These include the Sperm Chromatin Structure Assay (SCSA), the terminal deoxynucleotidyl transferase dUTP Nick End Labeling assay (TUNEL), the Sperm Chromatin Dispersion (SCD) (or variants), the Comet assay and the toluidine blue stain [11,15,66,67,68,69,70]. Different studies suggest that the extent of sperm DNA damage depends on the evaluation method. Khalil et al. [15] found a higher proportion of cryopreserved bull sperm showing DNA damage with the Sperm Chromatin Structure Assay (SCSA) compared to toluidine blue staining. Peris-Frau et al. [71] compared the ability of SCSA and a variant of the Sperm Chromatin Dispersion test (SCD) to evaluate sperm DNA damage, but only the latter method detected a higher proportion of cryopreserved ram sperm showing DNA damage after 180 min of incubation. These findings could indicate that the diverse methods used to assess chromatin integrity provide different information about chromatin damage. For this reason and given the complexity of sperm chromatin, more than one method should be employed to assess sperm chromatin damage during different stressful conditions, such as freezing–thawing. 

Several coding and non-coding RNAs, nuclear proteins and other epigenetics marks from sperm are delivered to the offspring together with the paternal genome [72]. In consequence, aside from DNA damage, changes in the relative abundance of RNAs, aberrant DNA methylation, abnormal histone modifications or improper chromatin compaction in sperm due to alterations in the nucleoprotein structure could have a severe impact on fertilization or embryogenesis [59,73,74]. While the effect of freezing–thawing on sperm DNA stability has been widely investigated, few studies in ruminants explored the influence of freezing–thawing on sperm epigenome. Messenger RNA (mRNA) carries the genetic code to translate proteins, but there are other RNAs termed non-coding RNAs (ncRNAs) that do not code for proteins. Both types of RNAs (mRNA and ncRNAs) have been found to modulate a variety of biological functions in sperm [36]. In addition, some ncRNAs are also involved in epigenetic regulation [75]. In consequence, variations in RNA transcripts during cryopreservation could adversely affect sperm integrity, functionality and its fertilizing potential or make the sperm vulnerable to epigenetic errors. Chen et al. [76] reported that cryopreservation modified in bull sperm the relative abundance of four ncRNAs involved in embryo development. More studies conducted in non-ruminant species revealed that cryopreservation induces diverse epigenetic changes. In horses, sperm cryopreservation increased global DNA methylation [77], whereas in boar sperm, freezing–thawing decreased the relative abundance of mRNAs as well as the protein levels of some genes associated with DNA methylation (DNMT3A, DNMT3B), histone modifications (JHDM2A, KAT8) and genomic imprinting (IGF2) [78]. Recently, a comparative transcriptome analysis of embryos obtained with fresh and cryopreserved stallion sperm revealed that several sperm mRNA transcripts with a relevant function in early embryo development were downregulated after cryopreservation [79]. However, there is a lack of similar studies in ruminant species. This clearly warrants further investigation and even deeper transcriptomics analyses between fresh and cryopreserved sperm using recent advances in transcriptome amplification and next-generation sequencing, which would improve our knowledge about the molecular mechanisms involved in sperm cryodamage.

## 3. Molecular Mechanisms Involved in Those Factors That May Affect Sperm Cryotolerance

It is well known that seasonal variations can alter the resistance of sperm to the freezing–thawing process in some species, known as sperm cryotolerance. Warmer seasons, specially summer, negatively affect the fertility rate of many ruminants due to the detrimental effect of heat stress on the female and male reproductive tract [80,81,82]. Moreover, since small ruminants are seasonal breeding animals, a more marked seasonality pattern has been found in these species compared to large ruminants, which is more evident in wild species than in domestics [83]. Martínez-Fresneda et al. [84] suggested that the seasonal fluctuations in sperm cryotolerance could be associated with changes in the membrane composition of sperm, occurring during spermatogenesis as a result of variations in the proliferative activity of germ cells and Sertoli cells among seasons. In line with this hypothesis, earlier studies found seasonal changes in the phospholipid, cholesterol and protein content of sperm [85,86]. Moreover, Westfalewicz et al. [86] reported that the majority of proteins that underwent changes in abundance during seasons came from seminal plasma. Several seminal plasma components, including diverse proteins, interact with sperm and become attached to the sperm surface after ejaculation, protecting the sperm membrane from cryodamage [87]. Those seminal plasma proteins whose abundance increased during the breeding season, were mainly involved in lipid metabolism regulation (ALB and CLU; bull sperm [86]), prevention of premature capacitation (SPZ1 and PLA2G7; bull sperm [86]) and protection against cold shock (RSVP14, RSVP20 and protein >10 kDa component; ram sperm [88,89]).

Other studies conducted in bull, ram and buck revealed that quantitative variations in seminal plasma and sperm proteins also appeared between males [90,91,92,93,94,95,96], which could explain in part the individual variability observed in sperm freezability [97,98]. Males are frequently categorized as good or bad freezers depending on their post-thaw sperm quality; however, molecular biomarkers could predict more efficiently sperm cryotolerance before freezing–thawing in different individuals [10]. Several proteins have been identified as potential freezability biomarkers in different ruminant species. Among them, proteins involved in energy metabolism, motility regulation and sperm membrane protection increased in abundance in males classified as good freezers (Table 1). These changes could explain the greater sperm viability, mitochondrial activity and motility of good freezers compared to bad freezers [90,91,92,93,94,95,99,100,101]. In this context, freezability biomarkers can be a useful tool to select those males with a superior sperm freezing resilience. But additional studies using recent advances in mass spectrometry technologies are required to identify a greater number of proteins that can be used as freezability biomarkers for a further accurate classification into good or bad freezers, especially in those ruminant species where studies are scarce. Moreover, a better characterization of all these proteins associated with sperm freezability differences would also aid in detecting proteins with a cryoprotective effect that could be incorporated into the freezing medium for enhancing sperm cryopreservation outcomes. Apart from variations in protein content, single nucleotide polymorphisms in different genes (e.g., *HSP70* in buck sperm, *PRNT* in ram sperm and *CFTR* in bull sperm) have been correlated with inter-individual differences in sperm freezability [102,103,104]. Whether these genetic variations are the reason for the different protein profile of good and bad freezers still need to be elucidated.

On the other hand, it is widely known that different sperm subpopulations with distinct kinematic, functional and morphometric characteristics coexist in the ejaculates [105,106,107], which leads to inter- and intra-male variations. The different steps of freezing–thawing process have been shown to alter the distribution of these kinematic and morphometric sperm subpopulations originally present in fresh ejaculates of ram, bull and buck [108,109,110]. These findings suggest that those fresh ejaculates with a higher subpopulation of sperm exhibiting a fast and progressive motility pattern as well as a low sperm head area could resist more the cryopreservation process. However, the molecular mechanisms underlying differences between sperm subpopulations still remain unclear during cryopreservation owing to the difficulties of analyzing separately the different sperm subpopulations present in the ejaculate with the traditional molecular approaches. A recent empirical study on bull sperm subpopulations obtained by a sperm selection procedure showed that the kinematic and functional differences between these subpopulations could be attributed to the different protein profiles detected in these subpopulations [111]. In this context, the use of computational and multiparametric flow cytometry seems to be a promising strategy to study multiple proteins simultaneously to their effects on sperm functionality at a single-cell level [112]. This approach would enhance our molecular understanding about the heterogeneous complexity of ejaculates, thereby contributing to identifying those sperm subpopulations with high cryotolerance.

Another factor that may affect sperm cryotolerance is the sperm source (epididymal or ejaculated). After ejaculation, sperm come into contact with seminal plasma, whereas epididymal sperm are exposed to epididymal fluid, whose composition differs from seminal plasma due to the lack of secretions from accessory sex glands. The interaction of sperm with a different surrounding medium seems to be the reason for such differences, resulting in changes in the lipid–protein composition of the plasma membrane, which in turn alters membrane stability and, ultimately, the sperm’s ability to withstand cryopreservation [113,114]. A comparative proteomics analysis in fresh ram sperm revealed that three proteins (EDIL3, BSP5 and LEG1) from seminal plasma were only present in ejaculated sperm but not in epididymal sperm, whereas four membrane proteins (SPADH2, PPP1R7, BDH2 and RNASE9) were more abundant in ejaculated than epididymal sperm due to their higher concentration in the seminal plasma [115]. However, the role of these proteins in the reproductive and cryopreservation process still needs to be further characterized. Recently, a deeper proteomics study on a non-ruminant species (boar) showed intriguing results about how variations in the proteome of epididymal and ejaculated sperm could affect the sperm cryotolerance [116]. Many of the differentially abundant proteins between these sperm sources were mainly involved in energy metabolism, structural activity, redox homeostasis, immune response and fertilization. Although some proteins from seminal plasma are able to prevent or revert the cold-shock damage on sperm [87,117], other proteins from seminal plasma have been negatively correlated with sperm preservation ability [118]. For example, binder of sperm proteins (BSP), which are one of the most abundant protein families in bull and ram seminal plasma, exert a different effect during cryopreservation in these species. In bull, BSP proteins promote sperm capacitation and have a beneficial role in sperm function but, at the same time, stimulate cholesterol and phospholipid efflux from the sperm plasma membrane [119]. Cholesterol molecules provide rigidity and stability to the sperm membrane. In consequence, the release of cholesterol molecules from the sperm membrane due to the action of BSP proteins decreases the sperm freezing resilience, which is detrimental to post-thaw sperm quality [119]. However, in ram sperm, these proteins had beneficial effects on post-thaw sperm quality, protecting ram sperm from cryodamage [120]. Clearly, further proteomics studies in different ruminant species are required to find out those proteins responsible for the differences found between epididymal and ejaculated sperm cryotolerance in each species.

## 4. Molecular Aspects of Those Novel Strategies to Reduce Sperm Cryodamage

Currently, there is a wide variety of extenders that can be used during sperm cryopreservation in different ruminant species (reviewed by [7,11,121,122,123,124]); however, not all of them offer the same protection against sperm cryodamage. Therefore, sperm cryotolerance and post-thaw sperm quality can be affected by the type of cryoprotectants, antioxidants and other components incorporated into the freezing medium as well as by their concentration [11,31,125]. Extenders usually contain various components (buffers, antibiotics, sugars, fatty acids, cryoprotectants, antioxidants and other substances) to efficiently protect sperm viability and fertility during cryopreservation [123]. Cryoprotectants protect sperm from ice crystal formation, osmotic and chemical stress. Such components can be classified into permeating and non-permeating, and both types of cryoprotectants are usually included in the extenders. Glycerol is the permeating cryoprotectant most commonly used in ruminants during sperm cryopreservation, while egg yolk is the non-permeating cryoprotectant. The former is cytotoxic beyond certain concentration and has been shown to alter in bull sperm some proteins associated with sperm–oocyte binding (IZUMO4), energy metabolism (PDB1, NUDFV2, NDPK7), cytoskeleton organization (CAPZB, ODF2) and ROS metabolism (SOD2), which may negatively affect sperm function [48]. For this reason, the combination of glycerol with non-permeating cryoprotectants (egg yolk, fructose, sucrose or trehalose) seems to be the best alternative to reduce glycerol concentration and its side effects in red deer [126], buffalo [127], bull [128], buck [129] and ram sperm [130]. Recently, a novel cryoprotective agent, carboxylated poly-L-lysine, has been used to reduce glycerol concentration in the freezing medium, enhancing in vivo fertility of cryopreserved buffalo and bull sperm [131,132]. Regarding non-permeating cryoprotectants, it has been reported that egg yolk also alters the proteome of ram sperm before cryopreservation [40]. Therefore, special attention should be payed to sperm-cryoprotectant interactions since these interactions may affect sperm cryopreservation outcomes. Additional studies should be conducted to elucidate whether glycerol and egg yolk exert the same impact on the sperm proteome of other ruminant species.

Another strategy for protecting sperm against cryodamage is the increment of the cholesterol membrane content prior to cryopreservation by adding cholesterol-loaded cyclodextrins (CLC) to the freezing medium. This treatment improves sperm membrane stability after incorporating exogenous cholesterol to the plasma membrane, which in turn enhances sperm cryosurvival, motility, mitochondrial activity and the number of sperm attached to zona pellucida, reducing at the same time cryo-capacitation and premature tyrosine phosphorylation [133,134,135,136]. The beneficial effects of CLC seem to be greater in those ejaculates with low freezability, at least in ram sperm [137]. Moreover, the addition of CLC to the extender attenuated in gazelle sperm the degradation of three proteins related to energy metabolism and cytoskeletal organization (CAPZB, HSP90A, PAGM2) during the freezing–thawing process compared to untreated sperm, which may explain the increased motility observed in CLC treated sperm [38]. Notwithstanding, the effectiveness of this strategy differs between studies and also among ruminant species, possibly because the optimal concentration still needs to be determined.

Supplementation of the freezing medium with antioxidants reduces the negative effects generated by the excessive ROS production during cryopreservation, which improves sperm cryosurvival. Antioxidants can be classified into enzymatic and non-enzymatic, and both types can be added to the freezing medium, yielding different results [5,138]. The former includes superoxide dismutase (SOD), glutathione reductase (GR), glutathione peroxidase (GPx) and catalase (CAT), while the latter includes reduced glutathione (GSH), vitamins, plant extracts (e.g., cinnamtannin B-1), minerals, amino acids, proteins and other exogenous compounds (e.g., resveratrol or quercetin) with antioxidant properties [123,139]. Recent studies investigated the addition of different nanoparticles to the freezing medium to overcome the main drawbacks that conventional antioxidants could present, like the low durability to harsh conditions [140,141,142,143]. Nanotechnology advances have contributed to the design of novel nano-compounds that possess antioxidant properties, such as selenium, zinc oxide and apoferritin containing gold-silver nanoparticles. Addition of selenium nanoparticles to semen extender enhanced viability, motility and chromatin integrity of cryopreserved bull sperm, obtaining greater in vivo fertility results [140]. Similar results were reported in cryopreserved ram sperm when selenium particles were added to the freezing medium [141]. These findings can be explained by the positive effect that selenium nanoparticles exert on GPx activity, reducing oxidative stress, lipid peroxidation and apoptosis. Supplementation of the freezing medium with selenium or zinc oxide nanoparticles also improved the post-thaw quality of dromedary camel sperm [142]. Both nanoparticles have the ability to enhance the activity of antioxidant enzymes (GPx and SOD), GSH and scavenge ROS. Such properties improved viability, membrane integrity and motility of cryopreserved sperm while decreasing apoptosis. Another nanoparticle recently designed to mimic SOD, CAT and GPx activity consisted in a silver–gold nanohybrid in apoferritin cage [143]. Enrichment of semen extender with apoferritin containing gold–silver nanoparticles resulted in a greater sperm viability and motility after thawing due to the reduction of ROS levels and apoptosis during cryopreservation. These studies reaffirm the potential of nanoparticles to protect sperm from oxidative damage during freezing–thawing, but further investigations should be performed in different ruminant species to avoid nanotoxicity on sperm cells.

Melatonin is another potential candidate to include in the freezing medium due to its protective effect against oxidative stress, which is dose-dependent [144]. The beneficial effects of melatonin on sperm cryopreservation rely on its powerful antioxidant property and its ability to stimulate the enzymatic activity of SOD, GPx and CAT [145]. A recent study in ram showed that melatonin improves mitochondrial oxidative phosphorylation of frozen–thawed sperm by suppressing mitochondrial permeability transition pore (MPTP) opening [146]. MPTP is a multi-component protein aggregate in mitochondria that has two main functions: regulation of oxidative phosphorylation for energy synthesis and induction of cell death when converted into a non-specific channel. When this complex is open, mitochondrial function declines and the apoptosis-inducing factor as well as some pro-apoptotic factors, such as cytochrome c, are released, initiating apoptosis [147]. Addition of melatonin to the freezing medium prevents a prolonged opening of MPTP during cryopreservation, which in turn increases ATP production, improving post-thaw sperm motility [146].

Proteomics studies on seminal plasma have greatly contributed to identifying those proteins with beneficial effects on sperm cryopreservation, facilitating the generation of recombinant proteins as a promising strategy for sperm cryopreservation. Recently, supplementation of the extender with recombinant seminal plasma proteins such as regucalcin (RGN), a recombinant peptide containing four FNII domains (TrxA-FNIIx4-His_6_) and serine protease inhibitor kazal-type 3 (SPINK3) have been shown to exert a cryoprotective effect on sperm [148,149,150]. A greater number of cryopreserved buffalo sperm were attached to zona pellucida when the freezing medium was supplemented with the recombinant RGN due to its positive effect on post-thaw sperm motility and acrosome integrity [148]. The beneficial effects of RGN may rely on its calcium-regulating and antioxidant functions. In frozen-thawed ram sperm, TrxA-FNIIx4-His_6_ has been shown to reduce cryo-capacitation, enhancing in vitro fertilization rates [149]. Another protein from seminal plasma that prevents and reverts cryo-capacitation is SPINK3. Zalazar et al. [150] reported that addition of heterologous SPINK3 before or after freezing–thawing decreased protein tyrosine phosphorylation and intracellular calcium influx in ram sperm. However, other sperm parameters related to sperm quality and fertility only improved when this recombinant protein was added after cryopreservation, probably due to the interaction of SPINK3 with extender components such as egg yolk before freezing, which may alter the ability of this protein to reduce sperm cryodamage [151].

Antifreeze proteins and glycoproteins are other cryoprotective elements that deserve special attention. These proteins, which are produced by some insects, Antarctic fishes, crustaceans, bacteria, fungi and microalgae, have the capacity to protect sperm membrane from cryodamage by preventing ice crystal formation [152]. Addition of antifreeze protein and glycoprotein type I to semen extender significantly increased post-thaw motility in ram sperm [153], whereas in bull, supplementation with antifreeze protein type I only improved the osmotic resistance of sperm during cryopreservation [154]. In buffalo, different studies showed that an antifreeze protein type III, two antifreeze glycoproteins and a recombinant antifreeze protein from beetle *Dendroides canadensis* enhanced sperm membrane integrity and motility after thawing when added to the freezing medium prior to cryopreservation [155,156,157].

Another alternative to avoid ice crystal formation during conventional freezing procedures includes vitrification. This simple and cost-effective technique involves a short pre-freezing equilibration and an ultra-rapid cooling rate using different buffer systems and concentrations of cryoprotectants for each species due to species-specific characteristics [3]. Arando et al. [158] reported that a short equilibration period at 5 °C before vitrification and dilution of ram sperm with a freezing extender containing sucrose improved post-thaw viability and motility, although these parameters were lower than in fresh samples. Another study in ram sperm showed that vitrification with egg yolk and glycerol at the lowest sperm concentration gave acceptable results in terms of viability and acrosome integrity in comparison to sperm vitrified with sucrose and glycerol [159]. However, vitrified ram sperm had a lower quality than sperm cryopreserved by conventional method. A similar situation was reported in mouflon sperm vitrified with sucrose [160]. On the contrary, vitrification of Iberian Ibex (*Capra pyrenaica*) sperm yielded a similar post-thaw quality and in vitro fertilization rate than sperm cryopreserved by conventional method [161]. Although major improvements are needed, vitrification could be extremely useful for preserving wild and endangered ruminant species under field conditions since sophisticated equipment is not required and the technique is relatively fast.

## 5. Concluding Remarks and Future Directions

Cryopreservation alters a variety of proteins and ARNs transcripts involved in relevant sperm functions, such as sperm motility, capacitation, fertilization and embryo development. Understanding the molecular damages caused by the freezing–thawing process is fundamental to protect these molecular elements and prevent or reduce those changes in sperm structure or function that negatively affect the reproductive performance. This is particularly relevant in ruminants since the majority of these species are more prone to suffer greater sperm cryodamage, requiring major improvements in the freezing–thawing process to obtain fertilization rates comparable to fresh sperm.

Advances in proteomics, transcriptomics and epigenomics technologies have provided new insights into the mechanisms underlying sperm cryodamage and those factors affecting sperm cryotolerance. This valuable information, which has been widely described in this review, is essential for the detection of potential biomarkers to predict more accurately sperm freezability as well as for the development of new strategies to enhance sperm cryopreservation outcomes. However, several challenges still need to be resolved. To address these issues, future studies on sperm cryopreservation should combine multiple technologies to investigate changes in sperm protein levels and location simultaneously to their effects on sperm functionality. In this sense, it would be interesting to combine high-throughput mass spectrometry with multiparametric, computational and imaging flow cytometry. The combination of these powerful technologies would offer a deeper insight into the molecular and cellular changes induced by the freezing–thawing process, facilitating data interpretation to improve sperm functionality and fertility of cryopreserved samples in different ruminant species. In addition, further transcriptomics and epigenomics studies are also required to increase our knowledge about how sperm cryopreservation affect different RNA transcripts and epigenetic-related genes involved in fertilization and embryo development in ruminant species.

On the other hand, supplementation of the freezing medium with novel cryoprotectants, antioxidants and other new components such as proteins or nanoparticles requires a further optimization to be an effective alternative to the commercial extenders currently used for cryopreservation of ruminant sperm. Therefore, further investigations with a wider range of concentrations, combinations of various novel cryoprotectants, antioxidants and proteins, and validation of post-thaw sperm fertility are needed.

## Figures and Tables

**Table 1:** Proteins that can be used as biomarkers to predict sperm freezability in ruminants due to their higher abundance in good freezers (GF) compared to bad freezers (BF).

Protein Name	Species	Origin/Source	Function during Cryopreservation	References
Seminal plasma protein PDC-109 (BSP-A1/A2)	Bull	Seminal plasma	Sperm membrane protection.	[101]
Acidic seminal fluid protein (aSFP)	Bull	Seminal plasma	Sperm membrane protection against lipid peroxidation.	[101]
Clusterin (CLU)	Bull	Seminal plasma	Sperm membrane protection.	[101]
Heat-shock protein 90 (HSP90)	Bull	Ejaculated sperm	Sperm motility regulation and protection against oxidative stress, thermal stress and apoptosis.	[92]
	Ram	Seminal plasma		[93]
26S proteasome non-ATPase regulatory subunit 2 (PSMD2)	Ram	Seminal plasma	Optimal cell organization by removing misfolded or damaged proteins.	[93]
Tripeptidyl-peptidase 2 (TPP2)	Ram	Seminal plasma	Protection from cryo-capacitation, increasing sperm longevity and fertility.	[93]
Transitional endoplasmic reticulum ATPase (VCP)	Ram	Seminal plasma	Positive regulation of mitochondrial membrane potential.	[93]
Sorbitol dehydrogenase (SORD)	Ram	Seminal plasma	Sperm motility regulation.	[93]
Chaperonin-containing t-complex polypeptide 1 (CCT)	Ram	Seminal plasma	Sperm membrane stabilization through an efficient protein folding.	[93]
Acrosome formation-associated factor isoform 2 (AFAF)	Bull	Ejaculated sperm	Preservation of acrosome integrity and viability.	[94]
Disintegrin and metalloproteinase domain-containing protein 2 (ADAM2)	Bull	Ejaculated sperm	Sperm membrane stabilization.	[94]
Aquaporin 3 (AQP3)	Bull	Ejaculated sperm	Protection against osmotic changes by controlling efficiently the flux of water and glycerol in the sperm membrane. Also involved in sperm motility.	[99,100]
Aquaporin 7 (AQP7)	Bull	Ejaculated sperm	Protection against osmotic changes by controlling efficiently the flux of water and glycerol in the sperm membrane. Also involved in sperm motility.	[99]
Aquaporin 11 (AQP11)	Bull	Ejaculated sperm	Protection against osmotic changes by controlling efficiently the flux of water and glycerol in the sperm membrane.	[90]
ATP synthase subunit beta, mitochondrial (ATP1B1)	Bull	Epididymal sperm	ATP synthesis through the electron transport chain, which explains the higher mitochondrial activity and motility of GF.	[91]
Fumarate hydratase, mitochondrial (FH)	Bull	Ejaculated sperm	Involved in energy metabolism, which explains the higher motility of GF.	[95]

## Abbreviations

TCP1	T-complex protein 1 subunit alpha
LOC101123268	Dolichyl-diphosphooligosaccharide-protein glycosyltransferase
RPN1	Dolichyl-diphosphooligosaccharide-protein glycosyltransferase subunit 1
HEXB	Beta-hexosaminidase subunit beta-like isoform X1
CSNK1G2	Casein kinase I isoform gamma-2 isoform X2
ICA	Inhibitor of carbonic anhydrase-like isoform X3
LOC101123216	Disintegrin and metalloproteinase domain-containing protein 20
ADAM2	Fertilin beta
TIMP-2	Tissue inhibitor of metalloproteinases 2
HSP70	Heat shock 70 kDa protein
GLUT	Glucose transporter
CLU	Clusterin
BSP5	Binder of sperm protein 5
BSP1	Binder of sperm protein 1
aSFP	Acidic seminal fluid protein
HSP4AL	Heat shock 70 kDa protein 4 L isoform C1
TRAP1	Heat shock protein 75 kDa, mitochondrial isoform X3
GPX4	Phospholipid hydroperoxide glutathione peroxidase
GPX5	Epididymal secretory glutathione peroxidase
CSNK2A2	Casein kinase II subunit alpha
SOD2	Superoxide dismutase 2
PRDX5	Peroxiredoxin 5
COX5B	Cytochrome c oxidase subunit 5B, mitochondrial
AK1	Adenylate kinase isoenzyme 1
NUDFV2	NADH dehydrogenase flavoprotein 2
ODPB2	Pyruvate dehydrogenase E1 component subunit beta, mitochondrial isoform 2
ACO2	Aconitate hydratase, mitochondrial
NDPK7	Nucleoside diphosphate kinase 7
GPI	Glucose-6-phosphate isomerase
ALDOA	Fructose-bisphosphate aldolase
GAPDH5	Glyceraldehyde-3-phosphate dehydrogenase, testis-specific
PGK2	Phosphoglycerate kinase 2
PGAM2	Phosphoglycerate mutase 2
PKM2	Pyruvate kinase M2
TPI	Triosephosphate isomerase
TEKT4	Tektin 4
ACTL7B	Actin-like protein 7B
HSP90	Heat shock 90 kDa protein
DNMT3A	DNA (cytosine-5-)-methyltransferase 3 alpha
DNMT3B	DNA (cytosine-5-)-methyltransferase 3 beta
JHDM2A	JmjC domain-containing histone demethylation protein 2A
KAT8	K(lysine) acetyltransferase 8
IGF2	Insulin-like growth factor 2
ALB	Bovine serum albumin
SPZ1	Spermadhesin 1
PLA2G7	Platelet-activating factor ace- tylhydrolase precursor
RSVP14	Ram seminal vesicle protein 14 kDa
RSVP20	Ram seminal vesicle protein 20 kDa
PRNT	Prion protein testis specific
CFTR	Cystic fibrosis transmembrane conductance regulator
EDIL3	EGF-like repeat and discoidon 1-like domain-containing protein 3
LEG1	Liver enriched gene 1
SPADH2	Spermadhesin Z13-like
PPP1R7	Protein phosphatase 1 regulatory subunit 7 trifunctional
BDH2	Bodhesin-2
RNASE9	Inactive ribonuclease-like protein 9
IZUMO4	Izumo sperm–egg fusion protein 4
PDB1	Pyruvate dehydrogenase E1 component subunit beta, mitochondrial precursor
HSP90A	Heat shock 90 kDa protein alpha
